# Alginate Use in Orthopedics and Peripheral Nerve Repair: A Systematic Review

**DOI:** 10.7759/cureus.72480

**Published:** 2024-10-27

**Authors:** Matthew T Eisenberg, Joshua W Hustedt

**Affiliations:** 1 Orthopedic Surgery, University of Arizona College of Medicine - Phoenix, Phoenix, USA; 2 Hand Surgery, University of Arizona College of Medicine - Phoenix, Phoenix, USA

**Keywords:** alginate, hydrogel, nerve, orthopedic surgery, repair

## Abstract

The use of alginate, a derivative of seaweed, has been proposed for multiple orthopedic indications. We aimed to review the current use of alginate in orthopedics and to focus on the future applications of alginate for peripheral nerve repair. A comprehensive literature search was performed to identify biomechanical, laboratory, animal, and human studies where alginate has been utilized for orthopedic or nerve repair indications. A systematic review of orthopedic indications was conducted for safety and efficacy, and a specific focus was placed on alginate for use in peripheral nerve repair and reconstruction. Thirty-two studies were identified. Alginate has a strong history and safety profile for usage in orthopedic surgery. Its primary usage has been for the repair of articular cartilage, although it has also been used for disc regeneration of the lumbar spine and for cushioning joints in osteoarthritis. The primary indication in peripheral nerve repair is to create an environment that encourages Schwann cell migration and repair in nerve injuries while blocking fibrotic scar tissue formation by inhibiting the activity of fibroblasts. Alginate hydrogel may serve as a potential conduit for nerve regeneration in nerve injuries with small to medium-sized gaps.

## Introduction and background

Alginate is a hydrogel that is naturally derived from seaweed. It consists of a linear polysaccharide copolymer of 1,4-linked β-mannuronic acid and α-glucuronic acid monomers [1]. It has been lauded for its ability to provide an ideal healing environment for mesenchymal stem cell lineages while preventing fibrosis from fibroblast infiltration [2,3]. It has been shown to have a strong biocompatibility and negligible inflammatory response when tested in vivo [4,5]. This has made it a target for usage in many orthopedic applications.

Alginate has been most commonly used in articular cartilage repair [6,7]. This has been done with and without cellular seeding [8]. Hishimura et al. utilized alginate alone and showed that it helped to regenerate articular cartilage at the site of donor tissue for an osteochondral autograft transfer system procedure [9]. Baba et al. utilized alginate hydrogel in addition to a bone marrow-stimulating approach to enhance articular cartilage repair in canines [10]. Due to its anti-inflammatory effects, other authors have proposed its usage for the reconstruction of the nucleus pulposus following discectomy for disc herniation and for use in knee osteoarthritis [11,12].

Yet one of the most pressing indications is its use in bridging nerve gaps after nerve injury. Evidence suggests that nerve gaps greater than 5 mm need to be “bridged” with either conduits, autografts, or allografts [13]. Traditionally, conduits have been used for gaps from 5 to 20 mm, with autograft and allograft utilized for gaps greater than 20 mm. Conduits have been shown to be efficacious in smaller gaps, are less expensive than allografts, and are less morbid than autografts [13]. However, in larger gaps, and particularly in mixed sensory and motor nerves, there remains controversy on the ideal graft.

Alginate has been proposed as a possible medium to bridge nerve gaps. The purported benefits of alginate are its ability to provide an environment that allows for Schwann cell proliferation and migration while blocking fibroblast scar tissue formation [14-16]. In addition, the gel does not form a tube and therefore can be utilized for branching nerves [17,18]. However, despite the purported benefits, there have been few clinical studies in humans to examine the use of alginate for peripheral nerve repair. We designed this systematic review to examine the overall safety of alginate in orthopedics and to specifically identify the usage of alginate for peripheral nerve repair.

## Review

Methods

This study was a systematic review of all currently published studies utilizing alginate for orthopedic and peripheral nerve indications. The review was conducted in accordance with the Preferred Reporting Items for Systematic Reviews and Meta-Analyses (PRISMA) guidelines. A comprehensive literature search was performed in PubMed, Embase, OVID Medline, Google Scholar, and Cochrane Library to identify studies in orthopedics and nerve surgery utilizing alginate. We included all studies discussing alginate usage including biomechanical, laboratory, animal, and human studies. We examined the evidence for usage in orthopedics to identify the safety and efficacy of alginate usage. Search terms included the following: “orthopedics”, “alginate”, “nerve”, and “repair”.

**Figure 1:**
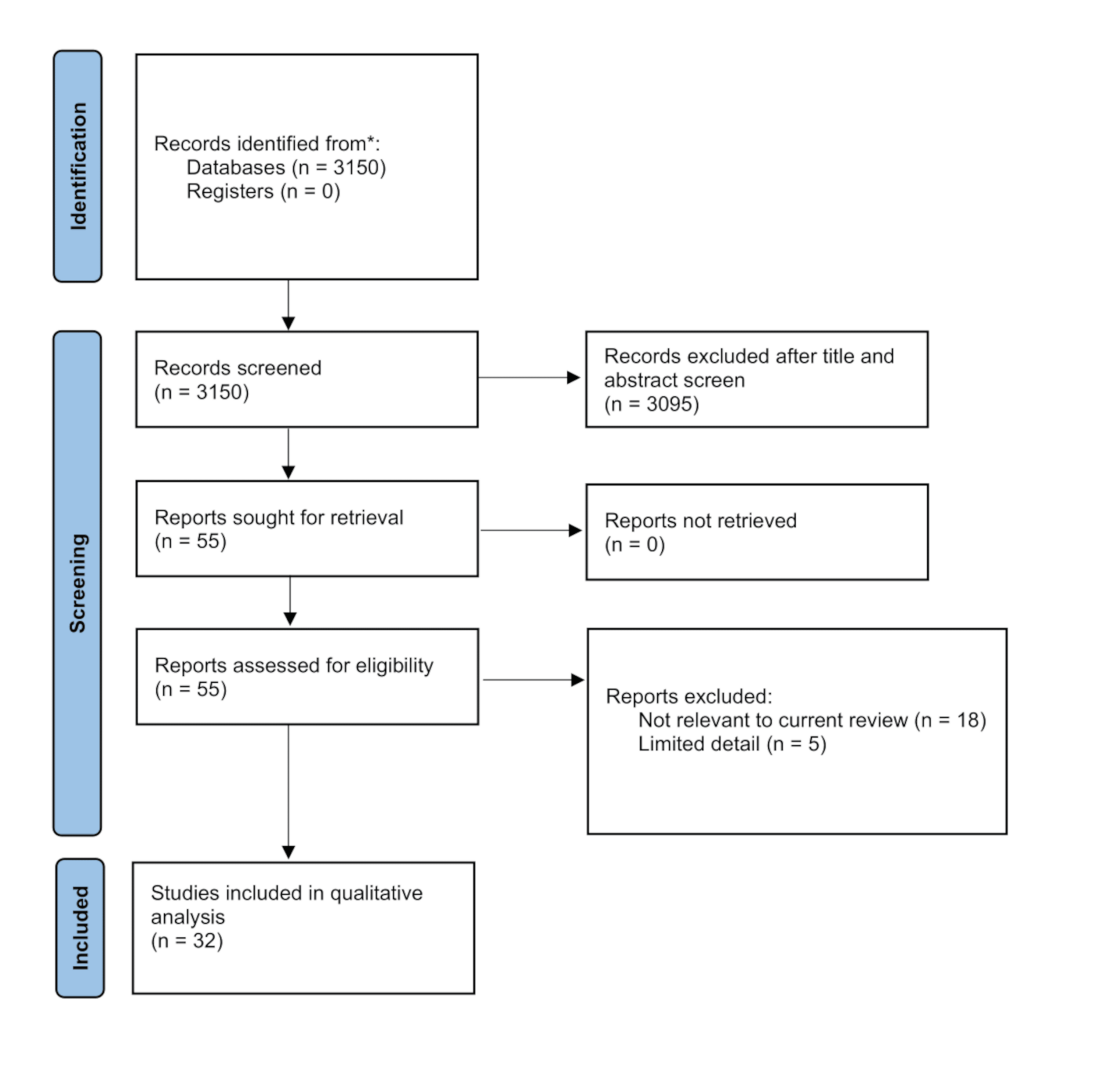
PRISMA flow diagram * PubMed, Embase, OVID Medline, Google Scholar, and Cochrane Library PRISMA, Preferred Reporting Items for Systematic Reviews and Meta-Analyses

The review was performed in duplicate by two reviewers. All abstracts were identified for possible inclusion, examining the possible usage of alginate in orthopedics and peripheral nerve repair. Studies were included if they were biomechanical, laboratory, animal, or human studies that discussed the use of alginate in orthopedics or nerve surgery. There were 3,150 citations initially identified across these databases. After initial screening, 3,095 were excluded, leaving 55 studies for full-text review. After a full-text review, an additional 23 studies were excluded, leaving a final number of 32 studies included in this review (Figure 1). For risk of bias assessment, the Newcastle-Ottawa questionnaire [19] was utilized for case-control and cohort studies, and the SYRCLE’s risk of bias tool was utilized for animal studies [20]. The included studies were grouped as “Articular cartilage regeneration,” “Lumbar disc regeneration,” “Knee osteoarthritis,” “Bleeding and scar tissue formation,” “Antibiotic delivery,” “Skeletal muscle regeneration,” “Cavernous nerve repair,” and “Peripheral nerve repair.”

Results

Study Characteristics

Thirty-two studies of alginate use in orthopedic surgery or peripheral nerve repair were identified. For the purposes of this review, studies were grouped by topic of research. Most included studies involved “Articular cartilage regeneration” (31.3%, n = 10), followed by “Peripheral nerve repair” (28.1%, n = 9), “Bleeding and scar tissue formation” (9.4%, n = 3), “Knee osteoarthritis” (9.4%, n = 3), “Cavernous nerve repair” (6.3%, n = 2), “Lumbar disc regeneration” (6.3%, n = 2), “Antibiotic delivery” (6.3%, n = 2), and “Skeletal muscle regeneration” (3.1%, n = 1). Most study designs utilized an animal model (71.9%, n = 23), while significantly fewer involved human subjects (18.8%, n = 6). The final three studies (9.4%) utilized a laboratory study design (Table 1).

**Table 1:** Studies utilized in this systematic review

Year	Author	Study design	Topic
2020	Onodera et al. [6]	Animal model	Articular cartilage regeneration
2012	Sukegawa et al. [7]	Animal model	Articular cartilage regeneration
2012	Igarashi et al. [8]	Animal model	Articular cartilage regeneration
2019	Hishimura et al. [9]	Animal model	Articular cartilage regeneration
2018	Baba et al. [10]	Animal model	Articular cartilage regeneration
2019	Kim et al. [21]	Animal model	Articular cartilage regeneration
2020	Kim et al. [22]	Animal model	Articular cartilage regeneration
2015	Baba et al. [23]	Animal model	Articular cartilage regeneration
2023	Onodera et al. [24]	Human study	Articular cartilage regeneration
2021	Momma et al. [25]	Human study	Articular cartilage regeneration
2021	Yamada et al. [11]	Human study	Lumbar disc regeneration
2021	Ura et al. [26]	Animal model	Lumbar disc regeneration
2012	Igarashi et al. [12]	Animal model	Knee osteoarthritis
2009	Almqvist et al. [27]	Human study	Knee osteoarthritis
2012	Dhollander et al. [28]	Human study	Knee osteoarthritis
2019	Ohta et al. [2]	Animal model	Bleeding and scar tissue formation
2005	Nagakura et al. [29]	Laboratory	Bleeding and scar tissue formation
2022	Singh et al. [30]	Animal model	Bleeding and scar tissue formation
2007	Ueng et al. [31]	Animal model	Antibiotic delivery
2000	Ueng et al. [32]	Laboratory	Antibiotic delivery
2010	Borselli et al. [33]	Animal model	Skeletal muscle regeneration
2006	Matsuura et al. [34]	Animal model	Cavernous nerve repair
2021	Narita et al. [35]	Animal model	Cavernous nerve repair
2002	Hashimoto et al. [14]	Animal model	Peripheral nerve repair
2005	Ohsumi et al. [15]	Animal model	Peripheral nerve repair
2001	Mosahebi et al. [16]	Laboratory	Peripheral nerve repair
2005	Hashimoto et al. [18]	Animal model	Peripheral nerve repair
2000	Suzuki et al. [36]	Animal model	Peripheral nerve repair
1999	Suzuki et al. [37]	Animal model	Peripheral nerve repair
2001	Sufan et al. [38]	Animal model	Peripheral nerve repair
2002	Wu et al. [39]	Animal model	Peripheral nerve repair
2016	Suzuki et al. [17]	Human study	Peripheral nerve repair

The results of the risk of bias assessment can be seen in Table 2 and Table 3. The study conducted by Yamada et al. [11] was not included as the results for this clinical trial have not yet been released. Finally, the 3 included laboratory studies [16,29,32] were not included in the risk of bias assessment as there is no universally accepted bias assessment tool for purely laboratory-based research.

**Table 2:** SYRCLE’s risk of bias tool for animal studies

	Author																						
Signaling question	Onodera et al. [6]	Sukegawa et al. [7]	Igarashi et al. [8]	Hishimura et al. [9]	Baba et al. [10]	Kim et al. [21]	Kim et al. [22]	Baba et al. [23]	Ura et al. [26]	Igarashi et al. [12]	Ohta et al. [2]	Singh et al. [30]	Ueng et al. [31]	Borselli et al. [33]	Matsuura et al. [34]	Narita et al. [35]	Hashimoto et al. [14]	Ohsumi et al. [15]	Hashimoto et al. [18]	Suzuki et al. [36]	Suzuki et al. [37]	Sufan et al. [38]	Wu et al. [39]
Was the allocation sequence adequately generated and applied?	Unclear	Unclear	Yes	No	Yes	No	Unclear	Unclear	Yes	Unclear	Yes	Unclear	Unclear	Unclear	Unclear	Unclear	Unclear	Unclear	Unclear	Unclear	Unclear	Unclear	Unclear
Were the groups similar at baseline, or were they adjusted for confounders in the analysis?	Yes	Yes	Yes	Yes	Yes	Yes	Unclear	Yes	Yes	Yes	Yes	Yes	Yes	Unclear	Yes	Yes	Yes	Yes	Unclear	Yes	Yes	Yes	No
Was the allocation adequately concealed?	Yes	Unclear	Unclear	No	Unclear	No	Unclear	Unclear	Unclear	Unclear	Unclear	Unclear	Unclear	Unclear	Unclear	Unclear	Unclear	Unclear	Unclear	Unclear	Unclear	Unclear	Unclear
Were the animals randomly housed during the experiment?	Unclear	Unclear	Unclear	Unclear	Unclear	Unclear	No	Unclear	No	No	Unclear	Unclear	Unclear	Unclear	Unclear	Unclear	Unclear	Unclear	Unclear	Unclear	Unclear	Unclear	Unclear
Were the caregivers and/or investigators blinded from knowledge of which intervention each animal received during the experiment?	Unclear	Unclear	Unclear	Unclear	Unclear	Unclear	No	Unclear	Yes	Unclear	Yes	Unclear	Unclear	Unclear	Unclear	No	Unclear	Unclear	Unclear	Unclear	Unclear	Unclear	Unclear
Were animals selected at random for outcome assessment?	Unclear	No	No	Yes	Unclear	Yes	Unclear	Yes	Yes	Unclear	Unclear	Unclear	Unclear	Unclear	Unclear	Unclear	Unclear	Unclear	Unclear	Unclear	Unclear	Unclear	Unclear
Was the outcome assessor blinded?	Yes	Yes	Yes	Yes	Yes	Yes	Unclear	Yes	Yes	Yes	Yes	Yes	Unclear	Unclear	Unclear	Unclear	No	Unclear	Unclear	Unclear	Unclear	Unclear	Unclear
Were incomplete outcome data adequately addressed?	Yes	Yes	Yes	Yes	Yes	Yes	Yes	Yes	Yes	Yes	Yes	Yes	Yes	Yes	No	Yes	Yes	Yes	Yes	Yes	Yes	Yes	Yes
Are reports of the study free of selective outcome reporting?	Yes	Yes	Yes	Yes	Yes	Unclear	Yes	Yes	Yes	Yes	Yes	Yes	Yes	Yes	Unclear	No	Unclear	Unclear	Unclear	Unclear	Unclear	Unclear	Yes
Was the study apparently free of other problems that could result in a high risk of bias?	Unclear	Unclear	Yes	Unclear	Yes	Unclear	Unclear	Unclear	Unclear	Unclear	Unclear	Unclear	Unclear	Unclear	Unclear	Unclear	Unclear	Unclear	Unclear	Unclear	Unclear	No	No

**Table 3:** Newcastle-Ottawa quality assessment tool for human studies

	Authors				
	Onodera et al. [24]	Momma et al. [25]	Almqvist et al. [27]	Dhollander et al. [28]	Suzuki et al. [17]
Selection					
Representativeness of exposed cohort	*	*	*	*	
Selection of non-exposed cohort					
Ascertainment of exposure	*	*	*	*	*
Demonstration that the outcome of interest was not present at the start of the study	*	*	*	*	*
Comparability					
Comparability of cohorts			*		
Outcome					
Assessment of outcome	*	*	*	*	*
Was follow-up long enough	*	*	*	*	
Adequacy of follow-up of cohorts	*	*	*		*
Total score (out of 9)	6	6	7	5	4

Discussion

This systematic review evaluated the use of alginate, a derivative of seaweed, in orthopedic surgery and in peripheral nerve repair. Thirty-two studies were identified. Most of the studies in orthopedics focused on the use of alginate to provide an ideal healing environment for mesenchymal stem cell-derived cells for tissue regeneration [6]. Alginate hydrogel was also suggested to deliver and/or maintain cells in a given area to aid in tissue regeneration [29]. Orthopedic indications primarily surrounded the use of articular cartilage regeneration but also included indications for lumbar discogenic pain and knee osteoarthritis [7,8,10,21-25]. Nerve indications included the repair of damaged nerves as well as the regeneration of the neural plexus in cavernous nerve repair [14-18,36-40].

Articular Cartilage Regeneration

Alginate has a long history of use for articular cartilage repair. It is commonly used for this indication based on its ability to protect cartilage cell precursors and allow them to isolate. One of the largest issues with cartilage repair is the creation of fibrocartilage after chondral drilling. Human bodies are not able to reform articular cartilage because of fibroblast infiltration. When fibroblasts infiltrate the cartilage repair site, they lay down scar tissue and create fibrocartilage, which is less dense and more friable than natural articular cartilage. Alginate hydrogels have been utilized to recreate the healing environment to create articular cartilage [6]. In addition, alginate hydrogels have been used as vehicle carriers to implant cartilage-producing cells and used for cartilage repair following autologous transfer and for meniscal repair [7,8,10,21-25].

Lumbar Disc Regeneration

One major benefit of alginate is its anti-inflammatory properties. Another is the ability to make alginate into a viscous product that can be injected. Both qualities have been highlighted in the intended use of alginate hydrogel for lumbar disc herniation [26]. Animal models have been created that suggest that alginate can be injected into a site of a void in the nucleus pulposus following a lumbar discectomy for disc herniation [26]. In this instance, the hydrogel has the potential to create an ideal healing environment for collagen-producing cells to recreate the nucleus pulposus as well as reduce the anti-inflammatory response of the nucleus pulposus cells. Injection of alginate hydrogel has also been proposed for the treatment of discogenic back pain as an anti-inflammatory agent. One human trial is underway for lumbar discogenic pain [11].

Knee Osteoarthritis

Due to the anti-inflammatory properties of alginate, there has been interest in utilizing it to treat osteoarthritis. Animal models have been conducted to show a reduction in knee pain and inflammation after alginate hydrogel injection in the knee. One model showed a reduction in intra-articular cellular inflammation with alginate injection [12]. This led the authors to suggest that alginate may be utilized as an injectable to reduce inflammation in knee osteoarthritis.

In a study conducted by Almqvist et al., they utilized alginate as a scaffold containing human mature allogenic chondrocytes for the treatment of osteochondral defects in the knee. They found statistically significant improvement in patient-reported outcome measures during a 24-month follow-up period. Adverse reactions were not observed [27]. Similar results were seen in a study conducted by Dhollander et al., which demonstrated improved Visual Analogue Scale scores after the implantation of alginate beads containing human mature allogenic chondrocytes [28].

Bleeding and Scar Tissue Formation

Alginate hydrogels have also been shown to inhibit fibroblast infiltration [29]. This is likely due to the large positive cationic charge of Ca2+ in the hydrogel. The cation charge prevents fibroblast migration into the gel and thereby prevents scar tissue formation. This allows the hydrogel to be utilized for a reduction in bleeding during surgery and to prevent postoperative fibrosis [29]. Animal models have shown the ability of alginate to reduce intra-peritoneal surgical site bleeding in a hepatectomy model as well as to significantly reduce postoperative fibrosis [2,30].

Antibiotic Delivery

Alginate has also been utilized as a delivery system for antibiotics and bone cells to treat infected bone defects. Ueng et al. utilized a rabbit model in which mesenchymal stem cells and vancomycin alginate beads were implanted into bony defects. The results of the study demonstrated sustained antibiotic elution for 14 days and osteogenic differentiation of the mesenchymal stem cells. As alginate is non-immunogenic and biodegradable, the authors of this study describe the potential benefit of alginate as an antibiotic delivery system as that there is no need for surgical removal of the beads. This contrasts with the current standard of using antibiotic-impregnated bone cement [31,32].

Skeletal Muscle Regeneration

Alginate hydrogels have also been used as a scaffold to deliver growth factors to ischemic skeletal muscle tissue. Borselli et al. utilized alginate to deliver VEGF and IGF1 to ischemic skeletal muscle tissue in a mouse model. The delivery led to nerve regeneration, angiogenesis, and skeletal muscle, which was grossly larger compared to controls [33].

Cavernous Nerve Repair

Researchers observing both the anti-fibrotic properties of alginate as well as its ability to provide an ideal healing environment for mesenchymal stem cells have hypothesized it may be an ideal medium for peripheral nerve repair. The thought process behind this is that nerve repair requires Schwann cells to proliferate and migrate in the absence of fibroblast fibrotic cellular components. Alginate seems to provide this environment, and it has therefore been utilized for peripheral nerve repair.

The area with the largest study of alginate is cavernous nerve repair. This repair occurs after a prostatectomy. Between 30% and 70% of patients experience erectile dysfunction following prostatectomy, even with advanced surgical techniques. This is due to damage to the cavernous nerve and the pre-sacral plexus that regulates the autonomic nervous system response required for erectile function. Multiple laboratory and animal trials have been conducted to show improvement in erectile function after prostatectomy with cavernous nerve repair with alginate nerve sheets [34,35].

Peripheral Nerve Repair

Peripheral nerve injury remains one of the most difficult surgical repairs conducted in hand and extremity surgery. Despite years of research, most peripheral nerve repairs still do not have satisfactory clinical results. Traditionally, repairs less than 5 mm are repaired directly. However, repair gaps of 5-20 mm are repaired with nerve conduits, and gaps greater than 20 mm are repaired with allograft or autograft [13]. All options have risks and benefits, including significant donor site morbidity for autograft and cost for allograft.

Alginate hydrogels have been proposed as the ideal healing environment for peripheral nerves as they allow the proliferation and migration of Schwann cells, the building blocks of peripheral nerve repair [14,16]. In addition, viscous injectable pure alginate sol has been shown to prevent fibroblast infiltration, thereby limiting the fibrosis of the nerve repair site [15]. The lack of scar and increase in Schwann cell activity may help increase nerve repair in gap reconstruction. In addition, the gel is not isolated to a tube and thereby can be utilized in areas of branching nerve reconstruction [18].

Much of the peripheral nerve regenerative work has been conducted in laboratory and animal models. Mosahebi et al. described the ability of alginate hydrogel to increase the proliferation and migration of Schwann cells in laboratory trials [16]. Hashimoto et al. further showed this ability for Schwann cell migration in rats [14]. Ohsumi et al. showed the ability of the hydrogel to prevent scar tissue formation in rat nerve repair [15].

Additional authors have conducted nerve reconstruction experiments in animal models. Suzuki et al. and Wang et al. showed the ability of alginate hydrogel to repair nerve gaps in rats [36,40]. Suzuki et al. and Sufan et al. also showed the ability of alginate hydrogel to bridge large sciatic nerve gaps in cats [37,38]. Wu et al. later showed the ability of an alginate sponge to bridge and repair a facial nerve gap in a cat [39].

To date, only one clinical trial has ever occurred in humans. This trial was done on two subjects in Japan. Suzuki et al. utilized an alginate sponge combined with a heparin-releasing compound and b-FGF [17]. The authors conducted two nerve repairs in traumatic lacerations. The first patient was a 57-year-old male who lacerated his thumb with an electric saw. The patient had a zone III distal thumb injury in which the proximal stump of the digital nerve could be identified but not the distal stump. The alginate sponge was placed on the end of the finger to help guide neural growth in a gap greater than 7 mm. His two-point discrimination (2PD) was 15 mm after surgery and improved to 5 mm at 6 months. The second patient was a 51-year-old man who fell snowboarding and lacerated his thumb. The patient sustained a 7-mm gap in the radial digital nerve that was treated with the alginate sponge. He had greater than 15 mm of 2PD after surgery, which improved to only 13 mm after six months [17].

Limitations

This study is not without limitations. Given the nature of systematic review, we are dependent on the availability and quality of the current published research. If relevant studies were missed or unpublished, this review may be incomplete or at risk of being affected by publication bias. Furthermore, there was significant heterogeneity in our studies in terms of design and topic of interest. Furthermore, the process of data extraction and assessment was subject to the authors’ interpretation and therefore could introduce bias into this systematic review.

## Conclusions

Alginate hydrogels have a long track record of safety when used in humans. Recent attention has been turned to the use of alginate in peripheral nerve repair due to its ability to provide an ideal healing environment for Schwann cell proliferation and migration. While ample evidence exists to suggest that alginate hydrogels may help improve nerve repair in animal models, evidence is mixed and lacking in humans. Additional clinical trials in humans are necessary to examine the safety and efficacy of nerve repair.
